# Health behaviors of German university freshmen during COVID-19 in association with health behaviors of close social ties, living arrangement, and time spent with peers

**DOI:** 10.1080/21642850.2021.1947291

**Published:** 2021-07-06

**Authors:** Chrys Gesualdo, Martin Pinquart

**Affiliations:** Department of Psychology, Philipps-University Marburg, Marburg, Germany

**Keywords:** University student, food consumption, physical activity, alcohol use, Covid-19

## Abstract

**Objective:**

The start of university is a critical period for health risk behavior (i.e. eating, physical activity, alcohol use) which can be influenced by expectations and by environmental factors such as living arrangement, health behaviors of close social ties (i.e. parents, partners, peers), and time spent with peers. We investigated associations between environmental factors and current/expected health behaviors of German freshmen during the COVID-19 pandemic.

**Method:**

A cross-sectional survey design was used. A total of *N* = 208 students (82.7% female; *M* age = 20.90, *SD* = 4.10) completed an online questionnaire assessing health behaviors and environmental factors at the beginning of their first semester.

**Results:**

Current and expected physical activity was associated to that of all social ties, current and expected alcohol use to partner's and peers’ alcohol use, while current and expected eating was only associated to peers’ eating. The relationship between partner's or peers’ and participant's alcohol use was moderated by coresidence, with a greater probability of engaging in these behaviors observed in case of coresidence. Perceived peer encouragement for alcohol consumption moderated the relationship between peer alcohol use and the number of drinks consumed by participants per month. Participants who spend more time with peers were more likely to consume higher amounts of alcohol. No differences were found regarding present and expected behaviors of participants who moved out of their parents’ home and those who did not.

**Conclusion:**

Partners and peers significantly influence students’ health behaviors, particularly alcohol use. Interventions to prevent health risk behaviors among freshmen should therefore address these social ties’ influence.

The start of university is often characterized by students’ increased experiences of autonomy and appears to be a critical period for health behavior alterations (Rozmus, Evans, Wysochansky, & Mixon, 2005). As first-semester students begin to experiment with their new lifestyle and freedom, alterations in health behaviors, such as unhealthy food consumption patterns (Racette, Deusinger, Strube, Highstein, & Deusinger, 2005), decrease in physical activity (Racette et al., 2005), and increase in alcohol use (Gfroerer, Greenblatt, & Wright, 1997) are likely to occur. Drawing from a cohort of German university freshmen, a large portion of students reported eating low amounts of fruits and vegetables, being physically inactive, and binge drinking (Keller, Maddock, Hannöver, Thyrian, & Basler, 2008). Moreover, readiness for behavior improvement was low, particularly for food consumption and alcohol use (Keller et al., 2008). Thus, health risk behaviors appear to be highly prevalent among German university students.

## Health behaviors and the transition to university

Evidence suggests that obesity rates in Germany are on the rise, particularly among young adults (Mensink et al., 2013). Individuals between the ages of 18 and 29 exhibit the highest percentages of obesity and overweight (Racette et al., 2005). Consistently, Mihalopoulos, Auinger, and Klein (2008) posit that the first year of university is a period of high risk for significant weight gain, and that first-year university students are almost 6 times more vulnerable for weight increases than the general population. Food consumption of university students have been consistently reported to be far from optimal, as low consumption of fruits, vegetables, and whole grains (Greene et al., 2011), and high consumption of saturated fat, refined carbohydrates, salt (Nelson, Lust, Story, & Ehlinger, 2009) and sugar-sweetened beverages (West et al., 2006) are common dietary patterns during the period of study. The alarming fluctuations in weight among university students can, in part, be attributed to unhealthy food consumption (Racette et al., 2005), which, if not modified, can predispose individuals to future obesity and health complications such as type 2 diabetes and cardiovascular disease (Hilger, Loerbroks, & Diehl, 2017). Additionally, during the trajectory of their undergraduate studies, students frequently do not meet health professionals’ recommended guidelines for physical activity, as physical inactivity typically increases during the first semester at university and continues to progress even after graduation (Racette, Deusinger, Strube, Highstein, & Deusinger, 2008). University students have reported that social influences, lack of willpower, lack of time due to their busy academic schedule, and their parents giving academic success priority over exercise serve as barriers to physical activity (Arzu, Tuzun, & Eker, 2006; Jajat, Sultoni, & Suherman, 2017). Regular physical activity has been associated with the prevention of musculoskeletal disorders, coronary heart disease, type 2 diabetes, osteoporosis, obesity and colon cancers (Vuori, 1995). Moreover, a study by Bray and Born (2004) demonstrated that students reporting higher levels of physical activity showed higher levels of vigor and lower levels of fatigue than those who were physically inactive. Thus, physical activity is a central protective factor against adverse health outcomes. Furthermore, problematic alcohol use among university students remains a fundamental health concern. In particular, the first semester at university often involves the initiation or an increase of alcohol use (Gfroerer et al., 1997). Motivations for drinking among first-year university students include ‘fitting in’, peer pressure, and regarding it as socially acceptable (Lindsay, 2006). Alcohol use among university students continues to increase, and detrimental consequences such as delinquency, sexual assault and memory blackouts are often reported (Rossow, Keating, Felix, & McCambridge, 2016). Alcohol use can affect students’ academic performance and health outcomes as it is reported to be a major predictor for early loss of health (Rossow et al., 2016).

## Environmental influences on health behaviors

The aforementioned health risk behaviors can be influenced by environmental factors such as the health behaviors of close social ties (i.e. parents, partner, and peers; Umberson, Crosnoe, & Reczek, 2010), time spent with peers (Barnes, Hoffman, Welte, Farrell, & Dintcheff, 2007), and changes in living arrangements (Small, Bailey-Davis, Morgan, & Maggs, 2013). Parents tend to have a strong impact on their offspring's health behaviors, with parental monitoring being one mechanism through which this is accomplished (Rozmus et al., 2005). Similarities in health behavior patterns of parents and their offspring have been found for healthy food consumption (El Ansari, Stock, & Mikolajczyk, 2012), physical activity (Yang, Telama, & Laakso, 1996), and alcohol use (Rossow et al., 2016). The start of university is preceded by a decline in parental supervision, which allows for increased accessibility of opportunities for risk behaviors (Rozmus et al., 2005). During adolescence, the influence of parents diminishes, and romantic partners and peers become significant social influences on an individual's health behaviors (Umberson et al., 2011). In line with this finding, associations between young adults’ health behaviors and the health behavior of their romantic partner have been demonstrated. For instance, Berge, MacLehose, Eisenberg, Laska, and Neumark-Sztainer (2012) found that young adults whose partner had health promoting behaviors in regard to food consumption and exercise were significantly more likely to eat healthy foods and to engage in physical activity than those whose romantic partners did not have health promoting attitudes and behaviors in regard to these health behaviors. Thus, having a partner who promotes healthy behavior appears to be a buffer against health risk behaviors. Furthermore, the norms and values present in the peer group have a significant influence on whether an individual engages in healthy behaviors or not (Umberson et al., 2010). As individuals become more independent from their parents, peers take a more prominent role and significantly influence an individual's immediate decisions (e.g. to drink at a party), and in this way, often promote health risk behaviors (Staff et al., 2010). As such, Barnes et al. (2007) posit that time spent with peers significantly contributes to problematic alcohol use among youth, whereas time spent with the family buffers said behavior.

Furthermore, the relocation from the parental home to enroll at university impacts food consumption patterns and physical activity, as it involves changes in the student's environment that can influence the availability of health promoting opportunities and the selection of these as opposed to unhealthy alternatives (Small et al., 2013). In a cross-sectional study involving university students in four European countries, El Ansari et al. (2012) found that students who lived with their parents consumed more fruits and vegetables than those who did not, suggesting parents’ influence on their offspring's food consumption habits. In regard to physical activity, a study found that youth living with parents reported higher levels of physical activity than those that did not live with their parents (Fan et al., 2019), whereas another study found the inverse (Jones, Harel, & Levinson, 1992). Furthermore, research showed that residence away from the parental home is a strong predictor of problematic drinking among college students, such as consuming more alcohol (Gfroerer et al., 1997). Similarities in food consumption, physical activity, and alcohol use have also been found among cohabiting couples (Monden, 2007). In a qualitative study using focus group discussions, university students in Europe stated that their close social ties influence their health decisions, and that living away from their parents’ residence regulates whether they behave in a health promoting manner or not (Deliens, Clarys, De Bourdeaudhuij, & Deforche, 2014). Thus, university students appear to foresee that their health behaviors are to be influenced by their place of residence and by the health behaviors of their close social networks. Role modeling and social support and encouragement may be mechanisms through which close social ties influence an individual's health behaviors (Jones et al., 1992; Liu et al., 2017).

In addition, popular media often depicts the time at university as a period in which unhealthy food consumption, physical inactivity and alcohol use are viewed as typical conducts, construing unhealthy behaviors as an anticipated component of the university experience (Reynolds, 2014). This common representation may cause incoming students to expect that their time at university ought to involve unhealthy behaviors and that these are justified by their status as a student (Silver, 1996). Deliens et al. (2014) postulated that university students perceive cohabitation with their parents as a barrier to health risk behaviors – a perception that may effectively prevent students from enacting health demoting behaviors. Moreover, expectations about intended future health behavior may impact present and future well-being as they can influence performance, effort, and persistence of behavior (Rief & Anna Glombiewski, 2017). Evidence exists demonstrating an association between university freshmen's alcohol expectations and actual drinking behavior (Werner, Walker, & Greene, 1993). That is, in the context of alcohol use, expectations have been shown to predict future drinking behavior among college students. Consequently, expectations may prompt first-semester students to deprioritize and postpone the implementation of healthy behaviors, overlooking the challenges of modifying deep-rooted habits and the negative consequences of years of cumulative unhealthy behavior (Harris, 2017).

## The present study

First-semester university students are continuously exposed to health risk behaviors, emphasizing the importance of further efforts to understand the factors that impact health behaviors among this population. Although evidence supports the increased vulnerability to unfavorable changes in health behaviors that first-semester students present (Rozmus et al., 2005), the role of social ties, living arrangement, and time spent with peers as health behavior determinants among this population has not been amply nor concurrently addressed in past research. The association of parental, partner and peer influence and university students’ health behaviors have not been simultaneously addressed, as past research has typically focused on investigating the impact of a single social tie. Simultaneous analysis of these factors will provide insights regarding which social tie is most strongly related to health behaviors. Furthermore, the topic of expected health behavior in regard to food consumption and physical activity in the transition to university has not been directly addressed in previous studies. As students are aware of health behavior alterations in this process of transition (Reynolds, 2014), it is likely that expectations will play a role in health behaviors occurring following this transition, and that they will also differ with regard to the changing living arrangement. Moreover, most studies investigating health behaviors among university students have been conducted in the United States, and limited research investigating health behavior patterns and determinants among university students in Germany exists (Keller et al., 2008) despite the alarming health risk behavior figures reported for this population in Germany. Based on the aforementioned findings, we assessed potential environmental correlates of and influences on current and expected health behaviors (i.e. food consumption, physical activity, number of alcoholic drinks consumed per month, and binge drinking) of first-semester university students in Germany and investigated whether discrepancies between current and expected future behavior exist. Namely, we investigated which participants present larger discrepancies and which expect to behave in a more healthy or unhealthy manner based on two predictors: changes in living arrangements and health behaviors of close social ties (i.e. parents, partners, and peers). First, we hypothesized that participants who moved out of their parents’ home to attend university would exhibit larger discrepancies between their present and expected health behaviors, as university students perceive residing with their parents as a barrier to health risk behaviors (Deliens et al., 2014), and report greater changes in health behaviors than those that did not move out of the parental home (El Ansari et al., 2012). Second, we hypothesized that the reported health behaviors of social ties and participant's current and/or expected health behaviors would be correlated, as an individual's social ties appear to significantly influence their health behaviors, although the magnitude of the influence of individual social ties may vary based on additional factors (Umberson et al., 2010). Third, we hypothesized that this correlation would be stronger if participants live with said social tie based on evidence suggesting that coresiding with social ties appears to influence an individual's health behavior (El Ansari et al., 2012; Monden, 2007). Additionally, we investigated whether social ties’ attempts to motivate participants’ health behaviors actually relate to participant's behaviors. Fourth, we hypothesized that social tie's efforts to motivate health behaviors would partially moderate the association between the health behavior of social ties and current health behavior of participants. Furthermore, we assessed whether time spent with peers relates to participant's health behaviors. Fifth, we hypothesized that participants who spend more time with their peers will display fewer current health promoting behaviors than participants who spend less time with their peers or who still live with their parents, as research posits that time spent with peers predicts health risk behavior among youth (Barnes et al., 2007), and that leaving the parents’ home is associated with higher substance use due to increased opportunities to experiment with alcohol (Gfroerer et al., 1997). Our study was conducted during the COVID-19 pandemic where students spent less time at university due to distance learning and had fewer opportunities for health risk behavior due to contact restrictions and to the closure of potential locations for said behaviors (e.g. bars and restaurants; Zusammen gegen Corona, 2020).

## Method

A cross-sectional survey design was used. Ethical approval was granted by the Ethics Committee at the University of Marburg, Germany. First-semester German university students who were at least 18 years of age were recruited via e-mails inviting them to participate. Data collection took place online during the first four weeks of participants’ first semester (i.e. November to December 2020). This timeframe was selected to measure participants’ health behaviors before spending a lot of time at university and being influenced by their new environment. Participants first read information about the study, completed a consent form, and answered the questionnaire. Anonymous coding was used to ensure participant privacy. As compensation, participants could choose to either receive university credit points or to participate in a gift card raffle.

### Measures

Participants received a link directing them to the self-report measures. For translation of the instruments from English to German, recommendations of Foster and Martinez (1995) were employed.

***Sociodemographic Characteristics.*** Questions inquired about age, sex (male, female, non-binary), relationship status (has a partner or does not have a partner), whether they moved out of their hometown to the town of their university, whether they moved out of their parents’ home to attend university, with whom they currently live with, and how many hours per day they spend with their peers. To protect participant anonymity by averting the risk of reidentifying individuals, we did not assess specific national or ethnic background.

***Healthy Eating.*** All seven items of the eating behavior category of the Centers for Disease Control and Prevention's National College Health Risk Behavior Survey (NCHRBS; Douglas et al., 1997) were imparted to assess food consumption (i.e. consumption of fruits and vegetables and of foods typically high in fat content and sugar). Four of the NCHRBS items assessed daily consumption of heathy foods (i.e. fruits, fruit juice, uncooked vegetables, and cooked vegetables) and three of the items assessed daily consumption of unhealthy foods (i.e. hamburgers, hot dogs or sausages, fried potatoes or potato chips, cookies, doughnuts or cake). Two additional items following the NCHRBS's format were incorporated to assess supplementary unhealthy food items relevant for German university students (i.e. pizza as well as sweets and chocolate). The questions were adapted to address how many times a day in the past month certain food items were consumed, with a Likert scale response format of 0 times a day, 1 time a day, 2 times a day, and 3 or more times a day. Higher scores were coded to denote lower levels of healthy food consumption. An internal reliability analysis on our data resulted in a Cronbach's coefficient of *α* = .79, indicating good reliability as per George and Mallery’s (2003) rules of thumb. Furthermore, rephrased versions of these nine items were also administered to assesses expected food consumption during the first semester at university. The expectation items showed an internal consistency of *α* = .73.

***Physical Activity****.* Four out of the five physical activity items of the NCHRBS (Douglas et al., 1997) were included to asses current physical activity. The items addressed how many times a week in the last month did participants perform vigorous or moderate physical activity, stretching exercises, strengthening exercises, and walking or cycling, with a Likert scale response format ranging from 0 to 7 times a week. Consistent with the average value of the response options, participants with higher scores were considered to display lower physical activity levels. The scale showed an acceptable internal consistency of *α* = .76 for our study. Moreover, rephrased formats of the four items were integrated to assess expected physical activity during the first semester at university. Expectancy items showed an acceptable internal consistency of *α* = .77.

***Alcohol Use.*** Three items were adapted from the Alcohol Use Disorders Identification Test (AUDIT; Saunders, Aasland, Babor, de la Fuente, & Grant, 1993) to inquire about drinking behavior. The first item assessed on how many days during the past month did the participant consume alcohol, with a free input response format in which zero was the minimum input possible and thirty-one was the maximum input possible. The second item assessed how many standard drinks does a participant drink on a drinking occasion (i.e. a typical day in which they consume alcohol), with a free input response format in which zero was the minimum response possible. Results of the first and second items were multiplied to obtain the number of drinks per month. The third item assessed how often do participants drink five (for males), four (for females) or more standard drinks in a drinking occasion (thus indicating binge drinking). Reponses had a Likert scale format and ranged from never, less than monthly, monthly, or weekly. Participants who reported that they consume five (for males), four (for females) or more drinks on a drinking occasion weekly were regarded as binge drinkers. Parallel formats of the three items were created to addressed expected drinking behavior during the first semester.

***Health Behaviors of Social Ties.*** Nine items assessing how often does each social tie (i.e. parents, partner, and peers) consumes healthy food, performs physical activity, and drinks alcohol were developed for our study. Health behaviors of the parental dyad and the peer group were assessed. We assessed each of the three health behaviors with one item per social tie in order to minimize assessment time. Participants who initially stated that they do not have a partner completed five items only assessing how often do parents and peers eat in a healthy way, perform physical activity, and consume alcohol. Possible responses ranged from 1 = very often, 2 = often, 3 = rarely, to 4 = never.

***Social Ties’ Efforts to Encourage Health Risk Behaviors.*** Nine items inquiring about social ties’ efforts to motivate health demoting behaviors were developed for our study. Single items assessed how often does a social tie (i.e. parents, partner, and peers) motivate the participant to eat unhealthy food, to be physically inactive, and to consume alcohol. Motivation deriving from the parental dyad and the peer group was assessed. Responses ranged from very often, often, rarely, to never. Participants who initially stated that they have a partner completed a total of nine items (i.e. three items per social tie). Participants who stated that they do not have a partner completed six items only (i.e. three items addressing parents and three items addressing peers).

## Statistical analysis

Data analysis was conducted using IBM SPSS Statistics version 27. An a-priori power analysis indicated a minimum sample size of 182 participants needed for identifying small effects with 80% power at an alpha level of .05. An independent *t*-test examined baseline differences between participants who moved out of the parental home and those who did not with regard to age. In addition, chi^2^ analyses were conducted to examine sex differences and whether the hometown is in Germany or abroad. Differences between current and expected health behaviors were computed. Normality tests were conducted. Taking into account how ANCOVAs are robust for non-normality especially when applied to larger samples (Hair, Black, Babin, Anderson, & Tatham, 2005), the decision was made to run this test also if the assumption of normality was rejected. An Alpha level of 5% was adopted to determine significant results. The first hypothesis was tested using ANCOVAs including discrepancies in current and expected health behavior as dependent variables and age as well as whether the hometown is in Germany or abroad as control variables. Bivariate correlation coefficients were calculated to test our second hypothesis. Furthermore, moderation analyses using multiple regressions were conducted to test our third and fourth hypotheses. Lastly, hierarchical regressions whilst controlling for covariates were computed to investigate our fifth hypothesis.

## Results

***Sociodemographic Characteristics.*** A total of *N* = 212 participants (82.1% female; *M* age = 21.24, *SD* = 6.15) participated in our study. After inspecting for outliers, *n* = 4 participants were excluded due to high rates of missing answers resulting in a final sample of *N* = 208 (82.7% female; *M* age = 20.90, *SD* = 4.10). Overall, *n* = 87 (41.8%) had a partner, *n* = 197 (94.7%) reported that Germany is their home country, *n* = 135 (64.9%) moved out of their hometown to the town of their university, and *n* = 143 (68.8%) moved out of their parents’ home to attend university. Moreover, *n* = 46 (22.12%) reported living with parents, *n* = 32 (15.38%) reported living with their partner, *n* = 107 (51.5%) reported living with peers, *n* = 22 (10.58%) reported living alone, and *n* = 1 (.5%) reported other living arrangement.

***Health Behaviors of Social Ties and Living Arrangement*.** Participants who moved out of their parents’ home to attend university were significantly younger (*M* = 20.17, *SD* = 2.10), *t*(70.35) = −2.88, *p* = . 005 than those who did not (*M* = 22.51, *SD* = 6.39). Moreover, participants who moved out of their parents’ home did not differ from participants who did not with regard to sex, *X*^2^ (2, *N* = 208) = 4.66, *p* > .05, but the groups differed with regard to whether their hometown is in Germany or abroad, *X*^2^(1, *N* = 208) = 5.28, *p* = .02. Thus, age and hometown location were included as covariates in our analyses. Contrary to our first hypothesis, no significant differences were found regarding present and expected food consumption of participants who moved out of their parents’ home and those who did not, *F*(1, 204) = .17, *p* = .68. Parallel results were found with regard to difference between present and expected physical activity *F*(1, 204) = .18, *p* = .67, difference between present and expected number of drinks consumed per month, *F*(1, 204) = .17, *p* = .68, and difference between current and expected binge drinking, *F*(1, 204) = .38, *p* = .54. Moreover, we tested whether the present and expected future levels of the health behaviors differed between those that moved out and those that did not and found no significant differences.

***Social Tie's Health Behaviors.*** Our second hypothesis was partially supported with the most support concerning peers and the least support concerning parents. Students’ current and expected physical activity levels were associated with the physical activity levels of their parents (*r* = .21, *p* = .01 and *r* = .20, *p* = .004 respectively). Furthermore, current and expected physical activity was associated to the physical activity levels of romantic partners (*r* = .31, *p* = .01 and *r* = .34, *p* = .01 respectively). Moreover, the current and expected number of drinks consumed by participants per month was associated to the partner's alcohol use (*r* = .40, *p* < .001 and *r* = .33, *p* = .01 respectively). The same was true for current and expected binge drinking (*r* = .27, *p* = .01 and *r* = .24, *p* = .02 respectively). In regard to peers, current and expected food consumption (*r* = .19, *p* = .01 and *r* = .18, *p* = .01 respectively), physical activity (*r* = .31, *p* < .001 for current, *r* = .23, *p* = .001 for expected), number of drinks consumed per month (*r* = .23, *p* = .001 for current, *r* = .26, *p* < .001 for expected), and binge drinking behaviors were significantly associated to their peers’ corresponding behaviors (*r* = .30, *p* < .001 for current, *r* = .34, *p* < .001 for expected). In contrast, there were no associations between student current and expected behavior and the related parental behavior with regard to food consumption (*r* = .02, *p* = .81 and *r* = −.12, *p* = .09 respectively), drinks per month (*r* = .07, *p* = .35 and *r* = .05, *p* = .51 correspondingly) and binge drinking (*r* = .09, *p* = .22 and *r* = .03, *p* = .65 correspondingly). Similarly, participant's current and expected food consumption and the food consumption of their partner were not significantly correlated (*r* = .15, *p* = .17 and *r* = .19, *p* = .09 respectively).

***Coresidence with Social Ties.*** Results for our third hypothesis are presented in Table 1 for current and Table 2 for expected health behaviors. Living with parents did not moderate the relationship between parent food consumption, physical activity, and alcohol use and participant current and expected food consumption, physical activity, number of drinks consumed per month, and binge drinking respectively. In contrast, coresidence with the partner moderated the relationship between the partner's alcohol use and the current number of drinks consumed by participants in a month and expected binge drinking, although no significant moderating effects emerged in the other domains. Lastly, coresidence with peers moderated the relationship between peer alcohol use and participant current binge drinking, but not the association between peers’ health behaviors and participant's current and expected health behaviors in the other domains. Follow-up analysis indicated that the association between participant health behavior and social tie behavior was stronger among those who live with the corresponding social tie (*β*'s ranged from .48 to .40 and *p*'s ranged from .001 to .01) than among those who do not (*β*'s ranged from .23 to .10 and *p*'s ranged from .59 to .13). Thereby, our third hypothesis was only partially supported.

**Table 1.** Associations of living with social ties and social tie's health behavior with students’ current health behavior (Multiple regression analysis).

	Participant HB: Current Eating			Participant HB: Current PA			Participant HB: Current # of Drinks Consumed Monthly			Participant HB: Current Binge Drinking		
	*B*	*SE B*	*β*	*B*	*SE B*	*β*	*B*	*SE B*	*β*	*B*	*SE B*	*β*
Parents’ HB	.05	.29	.01	1.53	.49	.21^a^	1.06	1.31	.06	.10	.09	.08
Living with Parents	−.13	.45	−.02	−.49	.99	−.04	−2.40	2.28	−.07	−.15	.15	−.07
Parents’ HB x Living with Parents	.95	.67	.10	1.82	1.17	.11	1.48	3.08	.03	.22	.21	.08
Δ*R*^2^ **Step 1**		.00			.05^a^			.01			.01	
Δ*R*^2^ **Step 2**		.01			.01			.01			.01	
Δ*R*^2^ **Total**		.01			.06^a^			.02			.02	
Partner's HB	.67	.50	.16	2.06	.68	.34^a^	8.93	1.98	.49^b^	.47	.16	.34^a^
Living with Partner	.53	.70	.08	.62	1.38	.05	−2.97	2.35	−.12	−.16	.20	−.09
Partner's HB x Living with Partner	−.47	.91	−.06	−1.50	1.39	−.12	−7.28	3.62	−.22^a^	−.39	.30	−.15
Δ*R*^2^ **Step 1**		.03			.10^a^			.17^a^			.08^a^	
Δ*R*^2^ **Step 2**		.03			.01			.04^b^			.02	
Δ*R*^2^ **Total**		.06			.11^a^			.21^b^			.10^a^	
Peers’ HB	.92	.31	.21^a^	2.64	.57	.31^b^	5.39	1.56	.23^a^	.47	.10	.30^b^
Living with Peers	−.12	.37	−.02	.28	.79	.02	6.27	3.29	.23	.75	.21	.41^a^
Peers’ HB x Living with Peers	−.59	.62	−.07	.21	1.14	.01	.87	3.12	.03	.44	.20	.25^a^
Δ*R***^2^ Step 1**		.04^a^			.10^b^			.10^b^			.13^b^	
Δ*R*^2^ **Step 2**		.01			.00			.00			.02^a^	
Δ*R*^2^ **Total**		.05^a^			.10^b^			.10^b^			.15^b^	

Note: *HB*: health behavior; *B*: unstandardized regression coefficient; *SE*: standard error. *β*: standardized regression coefficient. Step 1 assessed main effects and step 2 the interaction effect. Living with a particular social tie was represented as a dummy variable. Social tie's HB refers to the corresponding participant behavior. For the interaction, social ties’ HB and coresidence with said social tie were centered at their means. ^a^ *p* < .05, ^b^ *p* < .01.

**Table 2.** Associations of living with social ties and social tie's health behavior with students’ expected health behavior (Multiple regression analysis).

	Participant HB: Expected Eating			Participant HB: Expected PA			Participant HB: Expected # of Drinks Consumed Monthly			Participant HB: Expected Binge Drinking		
	*B*	*SE B*	*β*	*B*	*SE B*	*β*	*B*	*SE B*	*β*	*B*	*SE B*	*β*
Parents’ HB	−.49	.29	−.12	1.41	.49	.20^a^	.71	1.22	.04	.03	.09	.02
Living with Parents	−.03	.45	−.01	−.16	.98	−.01	−2.46	2.12	−.08	−.22	.16	−.10
Parents’ HB x Living with Parents	−.14	.67	−.01	.42	1.17	.03	−2.62	2.85	−.07	.01	.21	.01
Δ*R*^2^ **Step 1**		.01			.04^a^			.01			.01	
Δ*R*^2^ **Step 2**		.00			.01			.01			.00	
Δ*R*^2^ **Total**		.01			.05^a^			.02			.01	
Partner's HB	−.82	.31	−.32^a^	2.04	.63	.36^a^	8.24	2.19	.42^b^	.47	.16	.35^a^
Living with Partner	.13	.43	.03	.84	1.29	.07	−4.09	2.60	−.16	−.12	.19	−.07
Partner's HB x Living with Partner	−.03	.55	−.01	−1.15	1.20	−.10	−7.07	3.99	−.20	−.58	.29	−.23^a^
Δ*R*^2^ **Step 1**		.10^a^			.12^a^			.14^a^			.06	
Δ*R*^2^ **Step 2**		.00			.01			.03			.04^a^	
Δ*R*^2^ **Total**		.01^a^			.13^a^			.17^a^			.10^a^	
Peers’ HB	−1.21	.69	−.12	1.89	.58	.22	5.51	1.44	.25^b^	.53	.10	.33^b^
Living with Peers	−.42	.82	−.04	.19	.80	.02	3.09	3.04	.12	.48	.22	.26^a^
Peers’ HB x Living with Peers	2.18	1.38	.11	−.49	1.16	−.03	−1.89	2.89	−.08	.16	.21	.09
Δ*R*^2^ **Step 1**		.02			.05^a^			.10^b^			.15^b^	
Δ*R*^2^ **Step 2**		.01			.00			.00			.01	
Δ*R*^2^ **Total**		.03			.05^a^			.10^b^			.16^b^	

Note: *HB*: health behavior; *B*: unstandardized regression coefficient; *SE*: standard error; *β*: standardized regression coefficient. Step 1 assessed main effects and step 2 the interaction effect. Living with a particular social tie was represented as a dummy variable. Social tie's HB refers to the corresponding participant behavior. For the interaction, social ties’ HB and coresidence with said social tie were centered at their means. ^a^*p* < .05, ^b^*p* < .01.

***Social Ties’ Efforts to Encourage Health Risk Behaviors.*** Results for our fourth hypothesis are presented in Table 3. Efforts from parents and partners to motivate unhealthy behaviors did not moderate the size of any association between student behaviors and expectations with the related social tie health behavior. In contrast, encouragement from peers to consume alcohol moderated the relationship between peer alcohol use and the number of drinks consumed by participants in a month; nevertheless, other moderating effects of peer effort to motivate healthy or unhealthy behavior in the other domains were not found. Follow-up analyses indicated that the association between participant alcohol use and peer respective behavior was stronger among those whose peers encouraged them to drink (*β*'s ranged from .30 to .20 and *p*'s ranged from .06 to .01) than among those whose peers did not motivate them to drink (*β*'s ranged from .04 to −.30 and *p*'s ranged from .81 to .40). Thus, our fourth hypothesis was also partially supported.

**Table 3.** Associations of social tie's efforts to motivate unhealthy behaviors and social tie's health behavior with students’ current health behavior (Multiple regression analysis).

	Participant HB: Current Eating			Participant HB: Current PA			Participant HB: Current # of Drinks Consumed Monthly			Participant HB: Current Binge Drinking		
	*B*	*SE B*	*β*	*B*	*SE B*	*β*	*B*	*SE B*	*β*	*B*	*SE B*	*β*
Parents’ HB	−.37	.52	−.08	1.48	.51	.21	.34	1.29	.02	.08	.09	.06
Parents’ Motivation UB	−.35	.42	−.09	1.25	.81	.11	−7.62	2.57	−.23^a^	−.23	.18	−.11
Parents’ HB x Parents’ Motivation UB	−.09	.71	−.02	−.17	1.30	−.01	−1.82	3.13	−.05	−.04	.22	−.01
Δ*R*^2^ **Step 1**		.01			.06^a^			.07^a^			.02	
Δ*R*^2^ **Step 2**		.00			.00			.01			.00	
Δ*R*^2^ **Total**		.01			.06^a^			.08^a^			.02	
Partner's HB	.57	.46	.14	1.92	.66	.32^a^	6.95	1.97	.38^a^	.39	.16	.28^a^
Partner's Motivation UB	−.15	.46	−.04	.34	.92	.04	.11	1.65	.01	.01	.14	.01
Partner's HB x Partner's Motivation UB	−.12	.51	−.03	−.41	.78	−.06	−1.28	2.16	−.06	.04	.18	.03
Δ*R*^2^ **Step 1**		.02			.09^b^			.16^a^			.07^a^	
Δ*R*^2^ **Step 2**		.01			.01			.01			.01	
Δ*R*^2^ **Total**		.03			.10^b^			.17^a^			.08^a^	
Peers’ HB	.88	.31	.20^a^	2.65	.57	.31^b^	3.01	1.64	.13	.28	.11	.18^a^
Peers’ Motivation UB	.22	.24	.06	.14	.64	.02	−1.87	1.76	−.11	−.24	.12	−.21^a^
Peers’ HB x Peers’ Motivation UB	−.04	.35	−.01	.02	.86	.01	3.71	1.81	.21^a^	.14	.12	.12
Δ*R*^2^ **Step 1**		.04^a^			.10^b^			.12^b^			.17^b^	
Δ*R*^2^ **Step 2**		.00			.00			.02^a^			.01	
Δ*R*^2^ **Total**		.04^a^			.10^b^			.14^b^			.18^b^	

Note: *HB*: health behavior; *B*: unstandardized regression coefficient; *SE*: standard error. *β*: standardized regression coefficient. Step 1 assessed main effects and step 2 the interaction effect. Social tie's HB and social tie's motivation for health behavior refer to the corresponding participant behavior. For the interaction, social ties’ HB and their efforts to motivate healthy behaviors were centered at their means. ^a^*p* < .05, ^b^*p* < .01.

***Time Spent with Peers*.** No significant results were found for current food consumption and physical activity (see Table 4). Significant main effects of time spent with peers were found for current number of drinks consumed per month and for current binge drinking (see Table 4). Participants who spend more time with their peers are more likely to consume a higher number of drinks per month and to engage in binge drinking. Accordingly, our fifth hypothesis was supported only in regard to alcohol use.

**Table 4.** Associations of time spent with peers and living with parents with students’ current health behavior (Hierarchical regression analysis).

	Participant HB: Current Eating			Participant HB: Current PA			Participant HB: Current # of Drinks Consumed Monthly			Participant HB: Current Binge Drinking		
	*B*	*SE B*	*β*	*B*	*SE B*	*β*	*B*	*SE B*	*β*	*B*	*SE B*	*β*
Age	−.01	.05		.03	.10	.02	−.09	.22	−.03	−.02	.02	−.08
Home Country	.95	.91		1.40	2.02	.05	−1.38	4.27	−.02	.35	.30	.09
Time with Peers	−.01	.03	−.03	−.08	.07	−.09	.72	.14	.36^b^	.03	.01	.25^a^
Living with Parents	.01	.46	.01	.01	1.02	.01	−2.26	2.16	−.07	−.12	.15	−.06
Time with Peers x Living with Parents	.43	.08	.04	−.19	.18	−.08	−.29	.38	−.05	.03	.03	.09
Δ*R*^2^ **Step 1**		.01			.01			.02			.03	
Δ*R*^2^ **Step 2**		.01			.01			.13^b^			.05^a^	
Δ*R*^2^ **Step 3**		.01			.01			.01			.01	
Δ*R*^2^ **Total**		.03			.03			.16^b^			.09^a^	

Note: *HB*: health behavior; *B*: unstandardized regression coefficient; *SE*: standard error; *β*: standardized regression coefficient. Step 1 assessed main effects and step 2 the interaction effect. Home country was represented as a dummy variable with 1 indicating that the home country is Germany and 0 indicating that the home country is abroad. Time with peers referred to number of hours per week. Time with peers and living with parents were centered at their means to compute their interaction. ^a^*p* < .05, ^b^*p* < .01.

## Discussion

The present study investigated whether living arrangements, health behaviors of close social ties, close social tie's efforts to motivate behavior, and time spent with peers predict current and expected health behaviors of university freshmen. Our findings provide valuable insights regarding individual and environmental factors related to health behaviors and health-related expectations during the transition to university. Contrasting our first hypothesis, no significant differences were found in regard to present and expected food consumption, physical activity, number of drinks consumed per month, and binge drinking among participants who moved out of their parents’ home to attend university and those who did not. Our findings differ from previous evidence suggesting that, in part due to an increase of novel environmental influences, individuals who leave the parental home to attend university eat less healthy foods (El Ansari et al., 2012), are less physically active (Fan et al., 2019), and consume more alcohol (Gfroerer et al., 1997) than individuals who do not leave the parental home. As the majority of our sample was female, our results may deviate from previous studies as female first-semester university students tend to show less unhealthy behavior than their male peers (Olfert et al., 2019). Moreover, we speculate that the lack of significant differences may have been affected by restrictions in response to the COVID-19 pandemic as social distancing and closed bars and pubs reduced opportunities for some unhealthy behaviors at the university town (Zusammen gegen Corona, 2020). Thus, our findings may reflect an atypical transition to university characterized by reduced opportunities for significant alterations in health behaviors stemming from these governmental regulations (e.g. temporary closing of restaurants, fitness centers, bars and pubs). Consistently, evidence suggests that during the COVID-19 pandemic, university students’ food consumption habits have become healthier (Duong et al., 2020), physical activity increased (Romero-Blanco et al., 2020), and alcohol use decreased (White, Stevens, Hayes, & Jackson, 2020). Also, our sample may eventually exhibit worsened health behaviors as a result of leaving the parental home, but these changes may not happen early in the first semester (Small et al., 2013).

Partially in line with our second hypothesis, we found that students’ current and expected physical activity levels were significantly correlated to those of their social ties’ – current and expected alcohol use was significantly correlated to both the partner's and peers alcohol use, while current and expected food consumption was significantly associated to their peers’ food consumption. The size of these correlations was, on average, only small to moderate as individual behaviors are influenced by a large number of factors (Monden, 2007).

Regarding our third hypothesis, living with parents did not moderate the relationship between parent behavior and participant current and expected behavior. With regard to food consumption, this result may reflect limited number of joint meals and/or freedom of family members to select their preferred food, and parental attempts to accept or foster the autonomy of their offspring (Reicks et al., 2015), which in turn reduce similarities in food consumption. However, a main effect of parental physical activity was found. An intergenerational transmission of beliefs and preferences towards physical activity may be an explanation for this (Palmer, 2015), suggesting that parents may still have an effect on their offspring's behavior irrespectively of living or not living together. Nonetheless, this effect may fade after a longer period of residence away from the parental home (Palmer, 2015). Moreover, evidence suggesting that alcohol use typically happens outside the parental home and in the peer context (Gfroerer et al., 1997) can help explain the non-significant association between student drinking behavior and coresidence with parents. In regard to the romantic partner, we found that participants are more likely to consume alcohol with their partner if they live together. This result is in line with findings reporting similarities in health behaviors among cohabiting couples (Monden, 2007). Additionally, main statistical effects for partner food consumption, physical activity and alcohol use on participant expected food consumption, expected physical activity and expected number of drinks consumed per month were found. This may indicate that participants expect to become more similar to their partner over time by changing their own behaviors in response to the behaviors of the partner. Moreover, opportunities for joint food consumption, physical activity, and alcohol use can take place irrespectively of living together or not and often happen outside the home. In regard to peers, participants were more likely to binge drink with their peers if they live together. Regarding the other domains, statistical main effects were found indicating that participants are likely to eat, be physical active, and drink in a similar way as their peers irrespectively of living together or not. Thus, although coresidence provides increased opportunity for parallel health related behaviors, opportunities for joint behavior exist despite the absence of young people's coresidence with close social ties as long as they can easily meet.

Our fourth hypothesis was true only in regard to encouragement from peers to consume alcohol, which moderated the relationship between peer alcohol use and the number of drinks consumed by participants in a month. Our finding corroborates previous reports which consistently regard peer influence as a fundamental factor prompting drinking behavior among first-year university students (Borsari & Carey, 2006). Peers’ influence may evoke such behaviors as peers serve as socializing agents and as role models for first-year students, which in turn may lead students to adopt the behaviors and attitudes of their peers (Lau, Quadrel, & Hartman, 1990). Thus, modeling of behavior and direct encouragement may be processes though which students’ alcohol use is influenced by peers. Nevertheless, moderating effects of other social tie's efforts to motivate unhealthy behavior were not found. One explanation to this finding could be that parental influence on their offspring's health behaviors appears to weaken once they leave the parental home, and, although parents’ influence on their offspring's health beliefs may persist, these are not always conveyed in their behaviors and can be lessened through exposure to the beliefs and behaviors of other important social agents (Lau et al., 1990). Furthermore, we may have not found a significant statistical effect for partner influence as the test power for this analysis was low compared to our analysis of parental and peer influence. Thus, significant effects of partner influence may have been found with a higher number of participants with a partner. Lastly, effects of peer encouragement on participant health behavior may have been limited in our sample as opportunities for joint behavior were restricted due to Covid-19 regulations (Zusammen gegen Corona, 2020).

In regard to our fifth hypothesis, associations of time spent with peers and health behaviors were found with regard to the number of drinks consumed per month and binge drinking, so that participants who spend more time with their peers are more likely to consume a higher number of drinks per month and to binge drink than those who do not spent much time with their peers. Thus, in line with previous findings, peer influence on alcohol use appears to be stronger than peer influence on other health behaviors (Lau et al., 1990), and free time spent with peers can significantly contribute to problematic alcohol consumption among university students (Barnes et al., 2007). We speculate that homophily, or peer selection, plays a crucial role in the desirability to spend time with peers that have similar drinking behaviors (Mundt, Mercken, & Zakletskaia, 2012).

Several limitations in the present study make inferences about health behavior and health behavior determinants of first-year university students in Germany premature. First, although we intended to collect data during the first week of the first semester only, we had no choice but to extend our data collection period to ensure a higher number of participants. Thus, participants may have been already influenced by their new environment and may have adapted their behavior accordingly prior to completing the study. Second, most of our participants were female which could have affected the absolute levels of some behaviors, such as heavy drinking. Future research should focus on including an equivalent number of males and females to allow for the elaboration of generalizable inferences. Third, due to the COVID-19 pandemic, some environmental factors that would typically influence first-year students’ health behaviors may have played a smaller role, making our outcomes considerably less applicable for times independent of COVID-19. Thus, a reproduction of our study at a time when environmental opportunities for behavior alterations in the transition to university are more feasible (i.e. end of the COVID-19 pandemic) may provide results that are more comparable to those in previous findings. Fourth, we did not (yet) collect longitudinal data to investigate actual changes in health behavior and contrast these to students’ original expectations. Further studies investigating actual changes in behavior following the transition to university and throughout the course of the study period would allow for the establishment of a greater degree of accuracy on this matter. Fifth, data about participants’ perceptions of the health behaviors of their close social ties was collected rather than the actual health behaviors of close social ties. Participants could possibly be somewhat biased in their interpretation of social ties’ health behaviors in a way that inflates correlations between the two. Lastly, due to the larger number of hypotheses investigated, there is some risk that a particular statistical effect might have become significant by chance. Despite these limitations, our findings present significant implications for the understanding of factors that impact health behaviors among first-year German university students and provide novel insights regarding expectations in health behaviors during the transition to university. Nonetheless, future efforts are needed to further understand the influence of environmental factors and expectancies on health behaviors of German first-semester university students.

## Conclusion

The current study demonstrates that, at least during the COVID-19 pandemic, moving out of the parental home to attend university seems not to have a short-term effect on the differences between present and expected health behaviors of German university freshmen. Moreover, we conclude that current and expected alcohol use is associated to both the partner's and peers alcohol use. As such, coresidence with the partner or with peers appears to significantly influence university freshmen's alcohol use. Furthermore, encouragement from peers to consume alcohol influences the number of drinks consumed by students in a month. Consistently, students who spend more time with peers are more likely to consume a higher amount of alcohol than those who spend less time with peers. Thus, to develop successful interventions for preventing health risk behaviors among freshmen, particularly excessive alcohol use, interventionists should challenge the influence of partners and peers, and address participants’ health behavior expectations.

## Supplementary Material

Supplemental Material
