# Supplementary material for: Health behaviors of German university freshmen during COVID-19 in association with health behaviors of close social ties, living arrangement, and time spent with peers
Source: Health Psychol Behav Med. 2021 Jul 6;9(1):582–99. doi: 10.1080/21642850.2021.1947291 (PMC8266231; doi:10.1080/21642850.2021.1947291)
Supplement: Supplemental Material [file RHPB_A_1947291_SM5673.zip › Codebook.docx]

**Codebook**

Health behaviors of German first-semester university students in association with health behaviors of close social ties, living arrangement, and time spent with peers

Chrys Gesualdo and Martin Pinquart

Department of Psychology, Philipps-University Marburg, Germany

| Variable | Label | Answer Format | Source |
| --- | --- | --- | --- |
| Sex | Sex of the participant | -9= No answer  1 = Male  2 = Female  3 = Other | Demographic questions designed for our study |
| Age | Age of the participant | Free input | Demographic questions designed for our study |
| Partner | Whether the participant has a partner or not | -9 = No answer  1 = yes  2 = no | Demographic questions designed for our study |
| HomeCountry | Whether the home country is in Germany or Abroad | -9 = No answer  1 = In Germany  2 = Abroad | Demographic questions designed for our study |
| MovedtoUni | Moved to the town where the university is located | -9 = No answer  1 = yes  2 = no | Demographic questions designed for our study |
| MovedOut | Moved out of the parental home to attend university | -9 = No answer  1 = yes  2 = no | Demographic questions designed for our study |
| Residence | Participant’s current living arrangement | -9 = No answer  1 = With parents  2 = Shared apartment  3 = Student hall  4 = With partner  5 = Alone  6 = Other | Demographic questions designed for our study |
| ResidenceOther | Other living conditions‎/location | Free input | Demographic questions designed for our study |
| TimeUni | Time spent at the university town | -9 = No answer  1 = The entire week and most of the weekend  2 = Only during the week but not during the weekend  3 = Only some hours a day (e.g. for seminars, lectures)  4 = Almost never because I can study online | Demographic questions designed for our study |
| PeerTime_Week | Free time spent with peers during the week in hours per day | Free input | Demographic questions designed for our study |
| PeerTime_Weekend | Free time spent with peers during the weekend in hours per day | Free input | Demographic questions designed for our study |
| PeerTime_Total | Total time spent with peers during the week plus time with peers during the weekend | Not any | Demographic questions designed for our study |
| ParentTime_Week | Free time spent with parents during the week in hours per day | Free input | Demographic questions designed for our study |
| ParentTime_Weekend | Free time spent with parents during the weekend in hours per day | Free input | Demographic questions designed for our study |
| ParentTime_Total | Total time spent with parents during the week plus time with parents during the weekend | Not any | Demographic questions designed for our study |
| AloneTime | Time spent alone in hours per day | Free input | Demographic questions designed for our study |
| Fruits | How often per day in the past month did the participant consume fruits | -9 = No answer  1 = 3 Times or more  2 = 2 Times  3 = 1 Time  4 = 0 Times | NCHRBS  Items included: 7/7 items of the eating behavior category to assess consumption of fruits and vegetables and of foods typically high in fat content and sugar  Internal Consistency = .79 |
| FruitJuice | How often per day in the past month did the participant consume fruit juice | -9 = No answer  1 = 3 Times or more  2 = 2 Times  3 = 1 Time  4 = 0 Times | NCHRBS  Items included: 7/7 items of the eating behavior category to assess consumption of fruits and vegetables and of foods typically high in fat content and sugar  Internal Consistency = .79 |
| RawVeggies | How often per day in the past month did the participant consume raw vegetables | -9 = No answer  1 = 3 Times or more  2 = 2 Times  3 = 1 Time  4 = 0 Times | NCHRBS  Items included: 7/7 items of the eating behavior category to assess consumption of fruits and vegetables and of foods typically high in fat content and sugar  Internal Consistency = .79 |
| CookedVeggies | How often per day in the past month did the participant consume cooked vegetables | -9 = No answer  1 = 3 Times or more  2 = 2 Times  3 = 1 Time  4 = 0 Times | NCHRBS  Items included: 7/7 items of the eating behavior category to assess consumption of fruits and vegetables and of foods typically high in fat content and sugar  Internal Consistency = .79 |
| GreasyMeat | How often per day in the past month did the participant consume hamburgers, hot dogs, or sausages | -9 = No answer  1 = 0 Times  2 = 1 Time  3 = 2 Times  4 = 3 Times or more | NCHRBS  Items included: 7/7 items of the eating behavior category to assess consumption of fruits and vegetables and of foods typically high in fat content and sugar  Internal Consistency = .79 |
| FriesChips | How often per day in the past month did the participant consume potato fries or chips | -9 = No answer  1 = 0 Times  2 = 1 Time  3 = 2 Times  4 = 3 Times or more | NCHRBS  Items included: 7/7 items of the eating behavior category to assess consumption of fruits and vegetables and of foods typically high in fat content and sugar  Internal Consistency = .79 |
| CookiesCakes | How often per day in the past month did the participant consume cookies, cakes, or doughnuts | -9 = No answer  1 = 0 Times  2 = 1 Time  3 = 2 Times  4 = 3 Times or more | NCHRBS  Items included: 7/7 items of the eating behavior category to assess consumption of fruits and vegetables and of foods typically high in fat content and sugar  Internal Consistency = .79 |
| Pizza | How often per day in the past month did the participant consume pizza | -9 = No answer  1 = 0 Times  2 = 1 Time  3 = 2 Times  4 = 3 Times or more | Two additional items following the NCHRBS’s format were incorporated to assess supplementary unhealthy food items relevant for German university students (i.e., pizza as well as sweets and chocolate).  Internal Consistency = .79 |
| Sweets | How often per day in the past month did the participant consume sweets or chocolate | -9 = No answer  1 = 0 Times  2 = 1 Time  3 = 2 Times  4 = 3 Times or more | Two additional items following the NCHRBS’s format were incorporated to assess supplementary unhealthy food items relevant for German university students (i.e., pizza as well as sweets and chocolate).  Internal Consistency = .79 |
| UnhealthyEating | Current eating: Sum of all 9 eating behavior items where higher scores reflect unhealthier eating | Not any | Not any |
| Cardio | How often per week in the past month did the participant perform cardio exercises for at least 20 minutes | -9.00 = No answer  1 = 7 days  2 = 6 days  3 = 5 days  4 = 4 days  5 = 3 days  6 = 2 days  7 = 1 day  8 = 0 days | NCHRBS  Items included: 4/4 items of the physical activity category to assess how many times a week in the last month did participants perform vigorous or moderate physical activity, stretching exercises, strengthening exercises, and walking or cycling Internal Consistency = .76 |
| Stretching | How often per week in the past month did the participant perform stretching exercises | -9.00 = No answer  1 = 7 days  2 = 6 days  3 = 5 days  4 = 4 days  5 = 3 days  6 = 2 days  7 = 1 day  8 = 0 days | NCHRBS  Items included: 4/4 items of the physical activity category to assess how many times a week in the last month did participants perform vigorous or moderate physical activity, stretching exercises, strengthening exercises, and walking or cycling Internal Consistency = .76 |
| Strengthening | How often per week in the past month did the participant perform strengthening or muscle training exercises | -9.00 = No answer  1 = 7 days  2 = 6 days  3 = 5 days  4 = 4 days  5 = 3 days  6 = 2 days  7 = 1 day  8 = 0 days | NCHRBS  Items included: 4/4 items of the physical activity category to assess how many times a week in the last month did participants perform vigorous or moderate physical activity, stretching exercises, strengthening exercises, and walking or cycling Internal Consistency = .76 |
| Walking | How often per week in the past month did the participant walk or rode a bike for at least 30 minutes | -9.00 = No answer  1 = 7 days  2 = 6 days  3 = 5 days  4 = 4 days  5 = 3 days  6 = 2 days  7 = 1 day  8 = 0 days | NCHRBS  Items included: 4/4 items of the physical activity category to assess how many times a week in the last month did participants perform vigorous or moderate physical activity, stretching exercises, strengthening exercises, and walking or cycling Internal Consistency = .76 |
| UnhealthyPA | Current physical activity: Sum of all 4 physical activity items where higher scores reflect unhealthier physical activity behavior | Not any | Not any |
| DaysAU | ‎Number of days in the past month in which the participant consumed alcohol | Free input response format in which zero was the minimum input possible and thirty-one was the maximum input possible | AUDIT  Items included: 3/10 items were adapted to inquire about drinking behavior |
| Drinks | Number of standard drinks consumed by the participant in a single drinking occasion | Free input response format in which zero was the minimum response possible | AUDIT  Items included: 3/10 items were adapted to inquire about drinking behavior |
| HeavyDrinking | ‎Number of days in the past month in which the participant consumed alcohol multiplied by number of standard alcoholic beverages consumed by the participant in a single drinking occasion | Not any | Not any |
| BingeMale | Binge drinking in males: How often does a male participant consumes 5 or more standard alcoholic beverages per drinking occasion | -9 = No answer  1 = Never  2 = Less than monthly  3 = Monthly  4 = Weekly | AUDIT  Items included: 3/10 items were adapted to inquire about drinking behavior |
| BingeFemale | Binge drinking in females: How often does a female participant consumes 4 or more standard alcoholic beverages per drinking occasion | -9 = No answer  1 = Never  2 = Less than monthly  3 = Monthly  4 = Weekly | AUDIT  Items included: 3/10 items were adapted to inquire about drinking behavior |
| BingeDrinking | Current binge drinking (sum of binge drinking scores of males and females) | Not any | Not any |
| ParentEating | How often do the participant's parents consume healthy food | -9 = No answer  1 = Very often  2 = Often  3 = Seldom  4 = Never | Nine items assessing how often does each individual social tie (i.e., parents, partner, and peers) consumes healthy food, performs physical activity, and consumes alcohol were developed for our study |
| ParentPA | How often do the participant's parents perform physical activities | -9 = No answer  1 = Very often  2 = Often  3 = Seldom  4 = Never | Nine items assessing how often does each individual social tie (i.e., parents, partner, and peers) consumes healthy food, performs physical activity, and consumes alcohol were developed for our study |
| ParentAU | How often do the participant's parents consume alcoholic beverages (recoded answers for higher scores to show more unhealthy behavior) | -9.00 = No answer  1 = Never  2 = Seldom  3 = Often  4 = Very often | Nine items assessing how often does each individual social tie (i.e., parents, partner, and peers) consumes healthy food, performs physical activity, and consumes alcohol were developed for our study |
| PartnerEating | How often does the participant's romantic partner consume healthy food | -9 = No answer  1 = Very often  2 = Often  3 = Seldom  4 = Never | Nine items assessing how often does each individual social tie (i.e., parents, partner, and peers) consumes healthy food, performs physical activity, and consumes alcohol were developed for our study. Participants who initially stated that they do not have a partner completed five items only |
| PartnerPA | How often does the participant's romantic partner perform physical activities | -9 = No answer  1 = Very often  2 = Often  3 = Seldom  4 = Never | Nine items assessing how often does each individual social tie (i.e., parents, partner, and peers) consumes healthy food, performs physical activity, and consumes alcohol were developed for our study. Participants who initially stated that they do not have a partner completed five items only |
| PartnerAU | How often does the participant's romantic partner consume alcoholic beverages (recoded answers for higher scores to show more unhealthy behavior) | -9.00 = No answer  1 = Never  2 = Seldom  3 = Often  4 = Very often | Nine items assessing how often does each individual social tie (i.e., parents, partner, and peers) consumes healthy food, performs physical activity, and consumes alcohol were developed for our study. Participants who initially stated that they do not have a partner completed five items only |
| PeerEating | How often do the participant's peers consume healthy food | -9 = No answer  1 = Very often  2 = Often  3 = Seldom  4 = Never | Nine items assessing how often does each individual social tie (i.e., parents, partner, and peers) consumes healthy food, performs physical activity, and consumes alcohol were developed for our study |
| PeerPA | How often do the participant's peers perform physical activities | -9 = No answer  1 = Very often  2 = Often  3 = Seldom  4 = Never | Nine items assessing how often does each individual social tie (i.e., parents, partner, and peers) consumes healthy food, performs physical activity, and consumes alcohol were developed for our study |
| PeerAU | How often do the participant's peers consume alcoholic beverages (recoded answers for higher scores to show more unhealthy behavior) | -9.00 = No answer  1 = Never  2 = Seldom  3 = Often  4 = Very often | Nine items assessing how often does each individual social tie (i.e., parents, partner, and peers) consumes healthy food, performs physical activity, and consumes alcohol were developed for our study |
| ParentEating_Infl | How often do the participant's parents motivate the participant to consume unhealthy food | -9 = No answer  1 = Very often  2 = Often  3 = Seldom  4 = Never | Nine items inquiring about social ties’ efforts to motivate health demoting behaviors were developed for our study. Single items assessed how often does an individual social tie motivates the participant to eat unhealthy food, to be physically inactive, and to consume alcohol |
| ParentPA_Infl | How often do the participant's parents motivate the participant to not perform physical activities | -9 = No answer  1 = Very often  2 = Often  3 = Seldom  4 = Never | Nine items inquiring about social ties’ efforts to motivate health demoting behaviors were developed for our study. Single items assessed how often does an individual social tie motivates the participant to eat unhealthy food, to be physically inactive, and to consume alcohol |
| ParentAU_Infl | How often do the participant's parents motivate the participant to consume high amounts of alcoholic beverages | -9 = No answer  1 = Very often  2 = Often  3 = Seldom  4 = Never | Nine items inquiring about social ties’ efforts to motivate health demoting behaviors were developed for our study. Single items assessed how often does an individual social tie motivates the participant to eat unhealthy food, to be physically inactive, and to consume alcohol |
| PartnerEating_Infl | How often does the participant's romantic partner motivate the participant to consume unhealthy food | -9 = No answer  1 = Very often  2 = Often  3 = Seldom  4 = Never | Nine items inquiring about social ties’ efforts to motivate health demoting behaviors were developed for our study. Single items assessed how often does an individual social tie motivates the participant to eat unhealthy food, to be physically inactive, and to consume alcohol. Participants who initially stated that they do not have a partner completed nine items only |
| PartnerPA_Infl | How often does the participant's romantic partner motivate the participant to not perform physical activities | -9 = No answer  1 = Very often  2 = Often  3 = Seldom  4 = Never | Nine items inquiring about social ties’ efforts to motivate health demoting behaviors were developed for our study. Single items assessed how often does an individual social tie motivates the participant to eat unhealthy food, to be physically inactive, and to consume alcohol. Participants who initially stated that they do not have a partner completed nine items only |
| PartnerAU_Infl | How often does the participant's romantic partner motivate the participant to consume high amounts of alcoholic beverages | -9 = No answer  1 = Very often  2 = Often  3 = Seldom  4 = Never | Nine items inquiring about social ties’ efforts to motivate health demoting behaviors were developed for our study. Single items assessed how often does an individual social tie motivates the participant to eat unhealthy food, to be physically inactive, and to consume alcohol. Participants who initially stated that they do not have a partner completed nine items only |
| PeersEating_Infl | How often do the participant's peers motivate the participant to consume unhealthy food | -9 = No answer  1 = Very often  2 = Often  3 = Seldom  4 = Never | Nine items inquiring about social ties’ efforts to motivate health demoting behaviors were developed for our study. Single items assessed how often does an individual social tie motivates the participant to eat unhealthy food, to be physically inactive, and to consume alcohol |
| PeersPA_Infl | How often do the participant's peers motivate the participant to not perform physical activities | -9 = No answer  1 = Very often  2 = Often  3 = Seldom  4 = Never | Nine items inquiring about social ties’ efforts to motivate health demoting behaviors were developed for our study. Single items assessed how often does an individual social tie motivates the participant to eat unhealthy food, to be physically inactive, and to consume alcohol |
| PeersAU_Infl | How often do the participant's peers motivate the participant to consume high amounts of alcoholic beverages | -9 = No answer  1 = Very often  2 = Often  3 = Seldom  4 = Never | Nine items inquiring about social ties’ efforts to motivate health demoting behaviors were developed for our study. Single items assessed how often does an individual social tie motivates the participant to eat unhealthy food, to be physically inactive, and to consume alcohol |
| Fruits_Ex | How often per day this semester does the participant expect to consume fruits | -9 = No answer  1 = 3 Times or more  2 = 2 Times  3 = 1 Time  4 = 0 Times | Rephrased versions of all 9 items assessing eating behaviors (7 of which derive from NCHRBS were adapted to assesses expected eating behavior during the first semester at university  Internal consistency = .73 |
| FruitJuice_Ex | How often per day this semester does the participant expect to consume fruit juice | -9 = No answer  1 = 3 Times or more  2 = 2 Times  3 = 1 Time  4 = 0 Times | Rephrased versions of all 9 items assessing eating behaviors (7 of which derive from NCHRBS were adapted to assesses expected eating behavior during the first semester at university  Internal consistency = .73 |
| RawVeggies_Ex | How often per day this semester does the participant expect to consume raw vegetables | -9 = No answer  1 = 3 Times or more  2 = 2 Times  3 = 1 Time  4 = 0 Times | Rephrased versions of all 9 items assessing eating behaviors (7 of which derive from NCHRBS were adapted to assesses expected eating behavior during the first semester at university  Internal consistency = .73 |
| CookedVeggies_Ex | How often per day this semester does the participant expect to consume cooked vegetables | -9 = No answer  1 = 3 Times or more  2 = 2 Times  3 = 1 Time  4 = 0 Times | Rephrased versions of all 9 items assessing eating behaviors (7 of which derive from NCHRBS were adapted to assesses expected eating behavior during the first semester at university  Internal consistency = .73 |
| GreasyMeat_Ex | How often per day this semester does the participant expect to consume hamburgers, hot dogs, or sausages | -9 = No answer  1 = 0 Times  2 = 1 Time  3 = 2 Times  4 = 3 Times or more | Rephrased versions of all 9 items assessing eating behaviors (7 of which derive from NCHRBS were adapted to assesses expected eating behavior during the first semester at university  Internal consistency = .73 |
| FriesChips_Ex | How often per day this semester does the participant expect to consume potato fries or chips | -9 = No answer  1 = 0 Times  2 = 1 Time  3 = 2 Times  4 = 3 Times or more | Rephrased versions of all 9 items assessing eating behaviors (7 of which derive from NCHRBS were adapted to assesses expected eating behavior during the first semester at university  Internal consistency = .73 |
| CookiesCakes_Ex | How often per day this semester does the participant expect to consume cookies, cakes, or doughnuts | -9 = No answer  1 = 0 Times  2 = 1 Time  3 = 2 Times  4 = 3 Times or more | Rephrased versions of all 9 items assessing eating behaviors (7 of which derive from NCHRBS were adapted to assesses expected eating behavior during the first semester at university  Internal consistency = .73 |
| Pizza_Ex | How often per day this semester does the participant expect to consume pizza | -9 = No answer  1 = 0 Times  2 = 1 Time  3 = 2 Times  4 = 3 Times or more | Rephrased versions of all 9 items assessing eating behaviors (7 of which derive from NCHRBS were adapted to assesses expected eating behavior during the first semester at university  Internal consistency = .73 |
| Sweets_Ex | How often per day this semester does the participant expect to consume sweets or chocolate | -9 = No answer  1 = 0 Times  2 = 1 Time  3 = 2 Times  4 = 3 Times or more | Rephrased versions of all 9 items assessing eating behaviors (7 of which derive from NCHRBS were adapted to assesses expected eating behavior during the first semester at university  Internal consistency = .73 |
| UnhealthyEating_Ex | Expected eating: Sum of all 9 expected eating behavior items where higher scores reflect unhealthier eating | Not any | Not any |
| Cardio_Ex | How often per week in this semester does the participant expect to perform cardio exercises for at least 20 minutes | -9.00 = No answer  1 = 7 days  2 = 6 days  3 = 5 days  4 = 4 days  5 = 3 days  6 = 2 days  7 = 1 day  8 = 0 days | Rephrased versions of all 4 items assessing physical activity (all of which derive from NCHRBS) were adapted to assesses expected physical activity during the first semester at university  Internal consistency = .77 |
| Stretching_Ex | How often per week in this semester does the participant expect to perform stretching exercises | -9.00 = No answer  1 = 7 days  2 = 6 days  3 = 5 days  4 = 4 days  5 = 3 days  6 = 2 days  7 = 1 day  8 = 0 days | Rephrased versions of all 4 items assessing physical activity (all of which derive from NCHRBS were adapted to assesses expected physical activity during the first semester at university  Internal consistency = .77 |
| Strengthening_Ex | How often per week in this semester does the participant expect to perform strengthening or muscle training exercises | -9.00 = No answer  1 = 7 days  2 = 6 days  3 = 5 days  4 = 4 days  5 = 3 days  6 = 2 days  7 = 1 day  8 = 0 days | Rephrased versions of all 4 items assessing physical activity (all of which derive from NCHRBS were adapted to assesses expected physical activity during the first semester at university  Internal consistency = .77 |
| Walking_Ex | How often per week in this semester does the participant expect to walk or ride a bike for at least 30 minutes | -9.00 = No answer  1 = 7 days  2 = 6 days  3 = 5 days  4 = 4 days  5 = 3 days  6 = 2 days  7 = 1 day  8 = 0 days | Rephrased versions of all 4 items assessing physical activity (all of which derive from NCHRBS were adapted to assesses expected physical activity during the first semester at university  Internal consistency = .77 |
| UnhealthyPA_Ex | Expected physical activity: Sum of all 4 expected physical activity items where higher scores reflect unhealthier physical activity behavior | Not any | Not any |
| DaysAU_Ex | Number of days in a month in which the participant expects to consume alcohol this semester | Free input response format in which zero was the minimum input possible and thirty-one was the maximum input possible | Rephrased versions of the 3 AUDIT items were created to addressed expected drinking behavior during the first semester |
| Drinks_Ex | Number of standard drinks the participant expects consume this semester in a single drinking occasion | Free input response format in which zero was the minimum response possible | Rephrased versions of the 3 AUDIT items were created to addressed expected drinking behavior during the first semester |
| HeavyDrinking_Ex | Number of days in a month in which the participant expects to consume alcohol this semester multiplied by number of standard drinks the participant expects consume this semester in a single drinking occasion | Not any | Not any |
| BingeMale_Ex | Expected binge drinking in males: How often does a male participant expects to consume 5 or more standard drinks per drinking occasion this semester | -9 = No answer  1 = Never  2 = Less than monthly  3 = Monthly  4 = Weekly | Rephrased versions of the 3 AUDIT items were created to addressed expected drinking behavior during the first semester |
| BingeFemale_Ex | Expected binge drinking in females: How often does a female participant expects to consume 4 or more standard drinks per drinking occasion this semester | -9 = No answer  1 = Never  2 = Less than monthly  3 = Monthly  4 = Weekly | Rephrased versions of the 3 AUDIT items were created to addressed expected drinking behavior during the first semester |
| BingeDrinking_Ex | Expected binge drinking this semester (sum of binge drinking scores of males and females) | Not any | Not any |

*Note.* NCHRBS = Centers for Disease Control and Prevention's National College Health Risk Behavior Survey (Douglas et al., 1997); AUDIT = Alcohol Use Disorders Identification Test (Saunders, 1993).

**References**

Douglas, K. A., Collins, J. L., Warren, C., Kann, L., Gold, R., Clayton, S., Ross, J. G., & Kolbe, L. J. (1997). Results from the 1995 National College Health Risk Behavior Survey. *Journal of American College Health, 46*(2), 55-66. https://doi.org/10.1080/07448489709595589

Saunders, J. B., Aasland, O. G., Babor, T. F., de la Fuente, J. R., & Grant, M. (1993). Development of the Alcohol Use Disorders Identification Test (AUDIT): WHO Collaborative Project on Early Detection of Persons with Harmful Alcohol Consumption--II. *Addiction, 88*(6), 791-804. https://doi.org/10.1111/j.1360-0443.1993.tb02093.x
